# Supplementary material for: Spike mutations that affect the function and antigenicity of recent KP.3.1.1-like SARS-CoV-2 variants
Source: J Virol. 2025 Oct 13;99(11):e01423-25. doi: 10.1128/jvi.01423-25 (PMC12614646; doi:10.1128/jvi.01423-25)
Supplement: Supplemental figure legends — Legends for Fig. S1 to S5. [file jvi.01423-25-s0007.docx]

#### **Supplementary Figure 1. Design of KP.3.1.1 spike deep mutational scanning libraries**

**A.** Method for producing genotype-phenotype linked pseudovirus-based deep mutational scanning libraries as applied to the KP.3.1.1 spike. 293T cells are transfected with lentivirus helper plasmids, lentiviral backbone plasmids encoding the barcoded KP.3.1.1 spike variant library, and VSV-G expression plasmid to produce VSV-G pseudotyped viruses. The viruses are then used to infect 293T cells expressing reverse tetracycline transactivator (rtTA) at low multiplicity of infection (MOI, <0.01) so that only a single virus genome integrates in any given cell. Cells are then selected for successful transduction using puromycin. From selected cells, genotype-phenotype linked virus libraries are made by inducing spike expression using doxycycline and transfecting lentivirus helper plasmids. To quantify the presence of non-functional as well as functional spike variants present in the libraries, we also rescue VSV-G pseudotyped viruses from the same library cells by transfecting lentivirus helper plasmids and VSV-G expression plasmids. **B.** Number of targeted and successfully included mutations in each of the two independent libraries. Note that our library design primarily targeted mutations expected to be functionally tolerated, see text and Methods for details. **C.** Distribution of mutations per spike variant for each library. **D.** Correlation between the cell entry effects for all high-confidence measured mutations in both of the two independent libraries. Throughout the paper we show the average measurement across both libraries unless otherwise indicated.

#### **Supplementary Figure 2. Effects of mutations on ACE2 binding**

**A.** To measure effects of mutations to spike protein on ACE2 binding, deep mutational scanning libraries are incubated with monomeric soluble human ACE2 at multiple concentrations followed by infection of 293T-ACE2 cells expressing medium levels of ACE2. Library variants with mutations that increase ACE2 are better neutralized by soluble ACE2 compared to variants with mutations that decrease ACE2 binding. **B.** Correlation between mutation effects on ACE2 binding and cell entry; note it is only possible for our method to measure ACE2 binding for mutations that maintain at least modest levels of cell entry. **C.** SARS-CoV-2 spike structure with one RBD up in contact with ACE2 (PDB: 8IOU). Spheres show ACE2 distal RBD sites with strong effects on ACE2 binding as highlighted in **Fig. 3B**.

#### **Supplementary Figure 3. Neutralization of KP.3.1.1 pseudovirus by serum from the same individual collected pre- and post-exposure to a JN.1 descendant spike**

**A.** Neutralization curves for the sera from seven individuals analyzed in this study. Each plot shows sera from the same individual pre- and post-exposure to JN.1-descendant spike. Curves were measured using standard neutralization assays with KP.3.1.1 spike pseudotyped lentiviral particles. **B.** Neutralizing titers against KP.3.1.1 pseudovirus for sera pre- and post-exposure to JN.1-descendant spike calculated from the neutralization curves in A. **C.** Correlation between deep mutational scanning measured sera escape scores for pre- and post- JN.1-descendant spike exposed sera for each individual. Each point is a different mutation and shows the measured effect of that mutation on escape from each serum.

#### **Supplementary Figure 4. Effects of mutations to KP.3.1.1 spike on serum neutralization as measured by pseudovirus neutralization assay**

**A.** Correlation between deep mutational scanning measured escape scores and IC50 values measured using a standard pseudovirus neutralization assay for various KP.3.1.1 spike mutants. **B.** Fold change in IC50 values for different KP.3.1.1 spike mutants relative to the unmutated KP.3.1.1 spike for pre- and post-vaccination or infection sera, as measured using a standard pseudovirus neutralization assay. All mutations were measured for four sera except for V570W which was only measured for two sera.

#### **Supplementary Figure 5. Escape for BD55-1205, SA55 and VYD222 antibodies measured by yeast-based RBD versus pseudovirus-based full-spike deep mutational scanning**

**A.** Top logoplot shows effects of mutations at key sites on BD55-1205 antibody binding as measured using yeast-based deep mutational scanning of JN.1 RBD in previously published work by Jian et al (6). The height of the letter indicates binding escape for each mutation. The bottom logoplot shows mutations effects on neutralization by BD55-1205 as measured by KP.3.1.1 full-spile deep mutational scanning and is the same as in **Fig. 6**, with mutations colored according to their effect on ACE2 binding in the KP.3.1.1 full-spike pseudovirus deep mutational scanning. Sites where the parental amino acid differs between JN.1 and KP.3.1.1 backgrounds are labeled in both logoplots; sites labeled in just the bottom logoplot have the same parental amino acid in both JN.1 and KP.3.1.1. **B.** Same as A but for SA55 antibody. **C.** Same as A and B but for VYD222 antibody and with additional escape data for BA.2.86 RBD from Yuan et al (45) yeast-based RBD deep mutational scanning. No measurement was reported for site 502 for BD55-1205, and sites 502 and 505 for SA55 in the Jian et al RBD-only deep mutational scanning data. For BD55-1205 no measurements are available for sites 480 and 488 in full spike deep mutational scanning because all mutations at those sites are highly deleterious for cell entry.

#### **Supplementary Figure 6. Cloning of KP.3.1.1 deep mutational scanning library**

**A.** To produce plasmid library for deep mutational scanning using Golden Gate assembly, the KP.3.1.1 spike sequence was divided into 17 overlapping tiles. **B.** For each tile we computationally designed a pool of oligos containing all desired mutations. **C.** Designed oligos were ordered as a single-stranded DNA (ssDNA) oligo pool from which oligos belonging to each of the 17 tiles were amplified with primers containing the BsmBI restriction site. Unmutated spike sequences flanking each tile were also amplified. **D.** Golden Gate assembly was performed to assemble each tile pool and flanking spike sequences into a shuttle vector. **E.** Assembled spike sequences were amplified and barcoded in the same PCR reaction. Amplified and barcoded spike sequences were pooled equimolarly to make one library. **F.** The barcoded spike pool was cloned into a lentiviral vector using a HiFi reaction.
