# Supplementary material for: Spike mutations that affect the function and antigenicity of recent KP.3.1.1-like SARS-CoV-2 variants
Source: J Virol. 2025 Oct 13;99(11):e01423-25. doi: 10.1128/jvi.01423-25 (PMC12614646; doi:10.1128/jvi.01423-25)
Supplement: Supplemental table legends — Legends for Tables S1 and S2. [file jvi.01423-25-s0008.docx]

#### **Supplementary Table 1. Information about sera used in this study**

We used pre- and post-exposure sera from seven adults. The table indicates the last exposure (by vaccination or infection) and the days after this exposure that the “post-exposure” sera was collected. The “pre-exposure” serum from each individual was the last blood drawn prior to this final exposure, although individuals had different number of vaccinations or infections before this “pre-exposure” serum was collected. The table also indicates the last exposure before the “pre-exposure” serum collection. In all cases, the “post-exposure” serum was after a vaccination with the KP.2 spike or an infection in May-November of 2024, when JN.1-descendant variants dominated in Washington state where all the sera were collected (1).

#### **Supplementary Table 2. Antibody sequences**

Variable chain sequences for BD55-1205, SA55 and VYD222 antibodies. The complete expressed polypeptide sequences, including the human IgG1, lambda, or kappa constant sequences are provided. Heavy (HC) and light chains (LC) were expressed with murine Ig heavy chain V region 102 as an N-terminal export signal sequence.

1. UW virology COVID-19 dashboard [Internet]. 2023. Available from: <https://depts.washington.edu/labmed/covid19/#sequencing-information>
